# The value of targeting recombination as a strategy against coronavirus diseases

**DOI:** 10.1038/s41437-020-0337-5

**Published:** 2020-06-30

**Authors:** Enrique Santiago, Armando Caballero

**Affiliations:** 1 Departamento de Biología Funcional, Facultad de Biología, Universidad de Oviedo, Oviedo, Spain; 2 Centro de Investigación Mariña, Departamento de Bioquímica, Genética e Inmunología, Edificio CC Experimentais, Campus de Vigo, As Lagoas, Universidade de Vigo, Marcosende, 36310 Vigo, Spain

**Keywords:** Medical genetics, Population genetics

In a recent note, Jensen and Lynch (2020) remarked on the role of “mutational meltdown” as a strategy to restrict the expansion of COVID-19 in human populations. Mutational meltdown is a well-known population genetics process by which a population may become extinct due to the accumulation of deleterious mutations (Lynch and Gabriel 1990). As explained by Jensen and Lynch (2020), a sufficiently large increase of mutation rate may lead to a spiral of decline in fitness that eventually causes the extinction of the population. In a separate body of literature, virologists refer to that process as “lethal mutagenesis” in the context of drug-induced increase in viral mutation rate (Bull et al. 2007). Based on that concept, the development of drug therapies to induce errors during virus replication, either by lowering its RNA polymerase fidelity or by using base analogs, may become a promising strategy to fight viruses (such as SARS-CoV-2).

An additional issue related to the above argument is the role of recombination. Muller (1964) emphasized that in species devoid of recombination the accumulation of mutations is higher than in species with sexual reproduction. In the latter case, deleterious mutations can be assembled in the same genome by recombination, facilitating their efficient elimination. In contrast, in the absence of recombination, mutations accumulate without the possibility of reconstructing genomes with a mutation number lower than the minimum present in a given generation. This process, known as Muller’s ratchet, is a consequence of the action of purifying selection, which ultimately leads to a strong reduction in effective population size (*N*_*e*_); i.e., an increase of genetic drift and a consequent increase in the rate of fixation of combinations of deleterious mutations. Conceptually, *N*_*e*_ refers to the number of individuals that effectively contribute to descendants in the long term which, under selection, can be much smaller than the population census size *N*. Muller’s ratchet can be considered as part of the more general theory of “background selection,” which predicts the reduction of *N*_*e*_ by the action of purifying selection on linked sites (Charlesworth 2013) and applies to both sexual and asexual species.

**Fig. 1:**
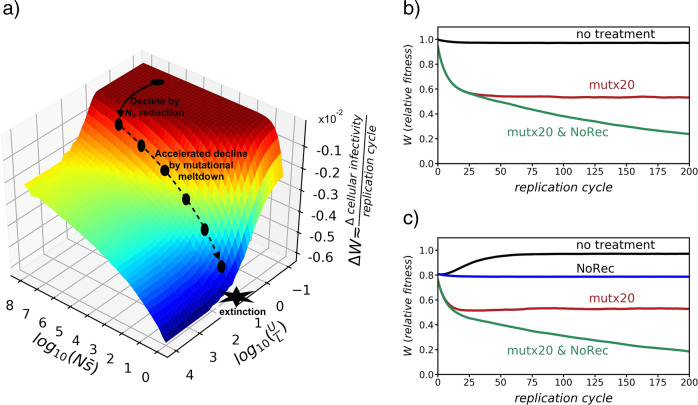
Evolution of fitness under deleterious mutation and recombination. **a** Change in the rate of relative fitness *W* (rate of cellular infectivity per replication cycle) at equilibrium calculated by equations given by Santiago and Caballero (2016) and represented as a function of the population size (*N*), the overall genomic rate of mutation (*U*), the average of deleterious effects ($\bar s$), here following an exponential distribution, and the degree of recombination, reflected by the genetic length (*L*) of the genome. The black dots indicate the position of a given population at different stages depending on its size and the selection, mutation, and recombination rates. (**b**, **c**) Change in fitness over replication cycles observed in stochastic simulations of populations with constant size *N* = 1000 and deleterious effect *s* = 0.1, assuming that the initial infection is caused by a single genotype free of deleterious mutations (**b**) or there is a coinfection of two genotypes with different deleterious mutations (**c**). Four scenarios are considered: No treatment *L* = 0.3, *U* = 0.03 (black line), a treatment implying a 20-fold increase in the mutation rate (mut × 20), a treatment implying absence of recombination (NoRec; only in panel **c**), and a treatment implying a 20-fold increase in the mutation rate and absence of recombination (mut × 20 & NoRec).

Santiago and Caballero (2016) showed that the rate of accumulation of deleterious mutations, extended to any degree of recombination, can be predicted from the effective population size theory of selection on linked sites and Kimura (1957) equations for the probability of fixation of mutations. Thus, the mean rate of decline in fitness (*W*) can be predicted (Fig. 1a) as a function of four factors: the population size (*N*); the average effect of deleterious mutations ($\bar s$); the rate of recombination, reflected here by the recombination map length (*L*) across the genome; and the overall genomic rate of deleterious mutations (*U*), the latter being proportional to the genome size and to the mutation rate per site. Coronaviruses have two characteristics that allow them to have the largest genomes among RNA viruses: an RNA polymerase with proofreading activity; and a homologous recombination mechanism associated with replication, which is effectively equivalent to sexual reproduction. These features entail a relatively low mutation rate and a high efficiency in eliminating deleterious mutations from the population of viral particles, which opens up the possibility of having large and sophisticated genomes. Although no direct measures of recombination rates are available for SARS-CoV-2, estimates from betacoronavirus mouse hepatitis virus (MHV) (Baric et al. 1995) suggest that, on average, they could be on the order of 10^−5^ between two consecutive sites per replication cycle. This quantity is approximately equivalent to a recombination map length of *L* = 0.3 Morgans, given that the genome of coronaviruses is about 30,000 nt long. On the other hand, mutation rates in MHV are on the order of 10^−6^ substitutions per site per replication cycle (Eckerle et al. 2010). Assuming that most mutations are deleterious or slightly deleterious, this is equivalent to a genomic rate of deleterious mutations probably close to *U* = 0.03.

As for any other successful population with an equivalent to sexual reproduction, coronavirus populations naturally stand at equilibrium somewhere on the upper plain of the surface of fitness changes in Fig. 1a. There is no steady decline of fitness on this plain, as virtually all deleterious mutations are eventually eliminated from the population. Given the above rates of mutation and recombination, the *U*/*L* ratio for coronaviruses could be about 0.1. The location on the surface in Fig. 1a is also determined by the product $N\bar s$, which is particularly important when *N* is transitorily small, as in the case of infection passage on a new host cell or individual (Duarte et al. 1992). However, for the moment, we shall consider that the product $N\bar s$ is sufficiently large to avoid any decline of fitness, and a feasible location for coronaviruses could be the black dot represented on the upper plain in Fig. 1a.

If the mutation rate *U* is increased, the equilibrium is pushed in the direction of the first arrow along the *U*/*L* axis to the second black dot, implying an acceleration in the rate of fitness decline of the population (in Fig. 1, given as infectivity rate) by accumulation of deleterious mutations. This accumulation is a consequence of the reduction of *N*_*e*_, but does not imply a reduction in population size *N* in the first stage. Then, as a consequence of the continuous accumulation of mutations, the population becomes trapped into a spiral of decline in fitness, which leads to a path of consecutive reductions of population size *N* in the direction of the second broken arrow along the $N\bar s$ axis, which eventually ends with the extinction, as explained by Jensen and Lynch (2020).

Recombination in coronaviruses makes the strategy of increasing the mutation rate less efficient than in the case of asexual reproduction. The success of this strategy depends on how close to the cliff the population is at equilibrium in Fig. 1a and how long the push is in the direction of the first arrow. Given that the location of the equilibrium point of the population on the plain depends on the ratio *U*/*L*, an increase in *U* would cause an equivalent effect to a similar decrease in *L*. The relevant point is that simultaneous changes in *U* and *L* have multiplicative effects on the displacement on the surface. In other words, an increase in the mutation rate may not be sufficient by itself to “knock down” the population, but if it is combined with the reduction in the recombination rate, the effect is amplified. Although it is unknown the extent to which both factors are functionally independent of each other in coronaviruses, it has been shown that some mutations can affect the rate of recombination without altering replication fidelity, for example, in polioviruses (Xiao et al. 2016).

While Fig. 1a represents equilibrium points of populations for particular combinations of parameters, Fig. 1b illustrates the temporal evolution of the absolute value of fitness over the infection process under three different scenarios: no treatment applied; a 20-fold increased mutation rate (mut × 20); and two treatments combined 20-fold increased mutation rate and complete restriction of recombination (mut × 20 & NoRec). The joint application of both treatments increases the magnitude of fitness drop when compared with the single treatment for mutation. The example assumes a constant population size of *N* = 1000 and constant deleterious effect *s* = 0.1, the latter being the average estimate for a variety of viruses (Sanjuán 2010). It is expected that *N* would be reduced over generations, accelerating the fitness decline. Thus, the example is a conservative one from that point of view. However, a substantially large deleterious effect is assumed for the purpose of illustration. Smaller mutational effects would imply a lower impact of both increased mutations and reduced recombination. In addition, other possible factors, such as advantageous mutations, compensatory mutations or epistasis are not considered in the model.

To date, no drug is known targeting the recombination process in coronaviruses and its development does not seem to be a priority either. However, the fact is that recombination rate in RNA viruses is determined by a variety of host factors affecting RNA metabolism, RNA silencing and the structure of the compartments where the viruses replicate (e.g. Garcia-Ruiz et al. (2018)). This evidence suggests that recombination processes could be feasible targets to develop new antivirals.

Recombination may also impact the spread of viruses across human populations through the effect of coinfections, as well known by epidemiologists (Balmer and Tanner 2011) and also documented for SARS-CoV-2 (Yi 2020). Coinfections by different genotypes are expected to result in the generation of new adaptive variants. In parallel, considering that most mutations are deleterious, the general principles described above can also be contemplated from an epidemiological perspective. To some extent, consecutive transmissions between community members result in increased load of deleterious mutations (Drop et al. 2020), but coinfections of the same cell by different strains could counteract that effect by allowing for the emergence of new genotypes with reduced mutational load. Here, the role of coinfections is equivalent to the effect of sexual reproduction on the accumulation of deleterious mutations. This is shown in Fig. 1c where two viral lineages are assumed to coinfect the host. The effect of recombination (no treatment) is a substantial recovery of the population fitness, which can be avoided if recombination is restricted or completely avoided. Thus, from Fig. 1c, it can be deduced that coinfection by different strains of a virus with substantial recombination implies an advantage for the virus and, thus, a high risk for the host.

In conclusion, our intention has been to point out the generally unnoticed issue that a combination of increased mutation and reduced recombination may produce an accelerated fitness decline of viruses in which recombination is not negligible. This is a point that may be further considered by virologists in order to look for new strategies to cope with viral proliferation.
